# Necroptosis-Related Genes Signatures Identified Molecular Subtypes and Underlying Mechanisms in Hepatocellular Carcinoma

**DOI:** 10.3389/fonc.2022.875264

**Published:** 2022-07-13

**Authors:** Jianguo Wei, Shuqian Hou, Minhua Li, Xiaofei Yao, Li Wang, Zhen Zheng, Haiqian Mo, Yu Chen, Xiaolu Yuan

**Affiliations:** ^1^ Department of Pathology, Shaoxing People's Hospital (Shaoxing Hospital, Zhejiang University School of Medicine), Shaoxing, China; ^2^ Department of Pathology, Maoming People’s Hospital, Maoming, China; ^3^ Department of General Medicine, Maoming People’s Hospital, Maoming, China; ^4^ School of Science, Wuhan University of Technology, Wuhan, China

**Keywords:** hepatocellular carcinoma, necroptosis, tumor microenvironment, immunotherapy, data mining

## Abstract

**Background:**

Although emerging evidence supports the relationship between necroptosis (NEC) related genes and hepatocellular carcinoma (HCC), the contribution of these necroptosis-related genes to the development, prognosis, and immunotherapy of HCC is unclear.

**Methods:**

The expression of genes and relevant clinical information were downloaded from TCGA-LIHC, LIRI-JP, GSE14520/NCI, GSE36376, GSE76427, GSE20140, GSE27150, and IMvigor210 datasets. Next, we used an unsupervised clustering method to assign the samples into phenotype clusters base on 15 necroptosis-related genes. Subsequently, we constructed a NEC score based on NEC phenotype-related prognostic genes to quantify the necroptosis related subtypes of individual patients.

**Results:**

We divided the samples into the high and low NEC score groups, and the high NEC score showed a poor prognosis. Simultaneously, NEC score is an effective and stable model and had a good performance in predicting the prognosis of HCC patients. A high NEC score was characterized by activation of the stroma and increased levels of immune infiltration. A high NEC score was also related to low expression of immune checkpoint molecules (PD-1/PD-L1). Importantly, the established NEC score would contribute to predicting the response to anti-PD-1/L1 immunotherapy.

**Conclusions:**

Our study provide a comprehensive analysis of necroptosis-related genes in HCC. Stratification based on the NEC score may enable HCC patients to benefit more from immunotherapy and help identify new cancer treatment strategies.

## Introduction

Primary liver cancer is one of the most advanced malignant tumors with poor prognosis, of which 80-95% are hepatocellular carcinoma (HCC) (1). Systemic therapy is the main treatment for advanced HCC, but due to significant molecular heterogeneity, protein kinase inhibitors targeting one or more sites have not shown the same significant therapeutic effect as lung cancer, colorectal cancer. Immune checkpoint blockades, represented by PD-1/PD-L1 inhibitors, have shown good therapeutic effects and are changing the therapeutic pattern of many tumors, including HCC. Nevertheless, it also faces many problems, such as primary drug resistance of tumor. The CheckMate-459 study showed that Nivolumab had an objective response rate of only 15% in the first-line treatment of HCC (2). Therefore, seeking effective biomarkers to screen patients for immunotherapy is the key to optimize the treatment strategy for HCC and improve the prognosis of patients.

Necroptosis is characterized by loss of plasma membrane integrity, swelling and deformation of cells and organelles, release of cellular contents, and further triggering inflammation to expand tissue damage (3, 4). In terms of mechanism, tumor necrosis factor receptor 1 (TNFR1), interferon receptor (IFNR), and Toll-like receptor 3/4/9 (TLR3/4/9) and DNA dependent activator of IFN regulatory factors were activated, thereby stimulating the signal to activate intracellular RIPK family kinases and initiating necroptosis (5–7). Increasing studies suggested that necroptosis contributes to the regulation of HCC oncogenesis (8). At present, chemotherapy drugs used to treat HCC generally inhibit tumor growth by inducing cell death. However, cell death resistance is the main reason for the unsuccessful treatment and recurrence of HCC, and most tumor cells are drug⁃resistant due to the dysregulation of apoptotic mechanism (9, 10). In Huh-7, HepG2, and Hep3B cell lines, necroptosis is inhibited due to genomic methylation near the RIPK3 transcription start site, and restoring the expression of RIPK3 can improve the sensitivity of cells to chemotherapy (11).

Recent evidence also indicated that necroptosis contributes to the regulation of cancer immunity (12). With the characters of both necrosis and apoptosis, necroptosis may trigger and amplify antitumor immunity in the immunotherapy of malignancy (13). Meanwhile, studies have shown that when tumor cells undergo necroptosis, IL-1α is released, which can activate dendritic cells. Activated dendritic cells produce cytotoxic factor IL-12 and activate CD8^+^T cells to induce anti-tumor immune response (5, 14). Similarly, DAMP from necrotizing tumor cells can induce strong expression of anti-tumor CD8^+^T cells (15). There is also evidence that NKT cells are involved in RIPK3-mediated immune responses against tumor cells, due to RIPK3 deletion impairs tumor activation by NKT cells (16).

In summary, necroptosis contributes to the regulation of HCC oncogenesis and cancer immunity. Although necroptosis is a promising tumor treatment target, the mechanism of necroptosis action in tumor needs to be further investigated. With the development of the Cancer Genome Atlas (TCGA), Gene Expression Omnibus (GEO) database, and immunotherapy dataset, big data mining has suggested as one of the promising ways to study the tumorigenesis mechanism and associated prognosis marker and therapy target of cancer. Herein, we mining database to investigate the expression profiles and prognosis significance of necroptosis-related genes in HCC, which may offer another evidence about the prognostic markers and molecular mechanisms in HCC.

## Materials and Methods

### Datasets and Preprocessing

The copy number variation (CNV), simple nucleotide variation (VarScan2 Variant Aggregation and Masking) were downloaded from TCGA database and UCSC Xena website, respectively. Next, 1398 HCC patients including RNA expression and corresponding clinical data were retrieved from TCGA (FPKM value, n=365) data portal, GEO database (GSE14520/NCI (17), n=221; GSE36376 (18), n=223; GSE76427 (19), n=115; GSE20140 (20), n=162; GSE27150, n=81), and International Cancer Genome Consortium (ICGC, n=231). In addition, a immunotherapy dataset (IMvigor210) with a total of 348 bladder cancer patients and corresponding clinical data were included (**Table 1**) (21). The FPKM value was firstly transformed to TPM to value more similar to those resulting from microarrays (22). Then, all raw data in the GEO database were microarray data processed on Affymetrix and Illumina. The raw data retrieved from the Affymetrix platform were processed using the RMA algorithm of the “affy” package in R for background adjustment and normalization (23). Finally, using “ComBat” algorithm of the “sva” package to batch effects among different datasets (24).

**Table 1:** Basic information of datasets included in this study for identifying distinct phenotypes.

Series accession numbers	Platform used	No. of input patients	Region	Survivval Outcome
GSE14520/NCI (GPL3921)	Affymetrix HT Human Genome U133A Array	221	USA	OS
GSE36376 (GPL10558)	Illumina HumanHT-12 V4.0 expression beadchip	223	Korea	OS
GSE76427 (GPL10558)	Illumina HumanHT-12 V4.0 expression beadchip	115	Singapore	OS
LIRI-JP	Illumina RNAseq	231		OS
TCGA-LIHC	Illumina RNAseq	365		OS
GSE20140 (GPL5474)	Human 6k Transcriptionally Informative Gene Panel	162	USA	OS
GSE27150 (GPL13128)	State Key Lab Homo sapien 2.6K	81	China	OS
IMvigor210	Illumina HiSeq 2000 RNAseq	348	USA	OS

### Identification of Differential Expression and Prognostic of Necroptosis-Related Genes

By reviewing the previous literature, we identified 76 necroptosis-related genes (**Supplementary Table 1**). The genes with the cut-off criteria of |log FC|≥ 1.0 and adj. P <0.05 were used as DEGs by using limma package (25). Using univariate Cox regression analysis to analyze the correlation between clinical information and necroptosis-related genes expression. Finally, 15 different and prognostic necroptosis related genes were identified.

### Genetic Mutation Analysis and Drug Sensitivity Analysis

Using “maftools” package, we then calculated mutation frequency of necroptosis-related genes. Moreover, “RCircos” package was used to visualized the chromosome location of necroptosis related genes CNV alteration. For drug sensitivity analysis, we collected the drug response data from the Genomics of Drug Sensitivity in Cancer (GDSC, https://www.cancerrxgene.org/) database (26) and Cancer Therapeutics Response Portal (CTRP, https://portals.broadinstitute.org/ctrp/?page=#ctd2BodyHome) database (27). The Spearman correlation analysis was performed using the “pRRophetic” package, which contained 138 drugs for analysis.

### Consensus Cluster Analysis

Based on 15 necroptosis-related genes identified above, we then conducted consensus cluster analysis with “ConsensusClusterPlus” package and cycle computation of 1,000 times as the threshold (28). The was followed by survival and gene-expression patterns analysis using “survival” package.

### Differentially Expressed Genes and Prognostic Genes Between the NEC Clusters

We identified two NEC clusters (NEC.cluster.A and NEC.cluster.B) base on 15 necroptosis-related genes. The DEGs was generated with “limma” package. The differential expression DEGs were assigned a |log2FC|>1 with adjusted P <0.05 as a significance threshold. Using univariate Cox regression analysis to analyze the correlation between clinical information and necroptosis related genes expression. Finally, 4000 different and prognostic genes were identified. Then, R package clusterProfiler was used to explore the functions between different and prognostic genes (adj. p< 0.05), and GO and KEGG enrichment analysis was performed.

### Construction of NEC Score

Next, we used principal component analysis (PCA) method to uantify the necroptosis related subtypes of individual patients (29). The model was based on the meta cohort and named the necroptosis score (NEC score). A NEC score for each patient was calculated according to the following formula:


$$\text{NEC score}=\sum PC1_{i}+PC1_{2}$$

where i is the TPM value of each screened gene.

### Analysis of Gene Set Enrichment

Gene set enrichment analysis (GSEA) is a computational method verifying whether *a priori* presetting of genes indicates statistically significant, concordant differences between two biological states (30). In order to explore the biological pathways involved in HCC progression, we divided into a NEC score.low group (n= 556) and a NEC score.high group (n= 599) based on the median of NEC score as a cut-off point and the number of permutations was 1000.

### Immune Cell Infiltration Analysis

ssGSEA method, which uses gene expression profiles to infer the number of tumor infiltrating immune cells (31). A series of analysis on the NEC score in HCC and its correlation with the abundance of immune infiltrates was performed.

### Immunohistochemistry Staining

8 HCC patients tissues and corresponding adjacent tissues were collected from the second affiliated hospital of Guangdong Medical University to explore the expression of 15 marker genes in the tissue samples. IHC was performed on formalinfixed, paraffin-embedded tissue sections using a two-step protocol. Briefly, paraffin section of 15 patients were first deparaffinized and heated in pressure cooker for 10 min. After cooling to room temperature, the sections were immersed for 5 min in PBS three times and followed by endogenous peroxidase activity blocking with 3% H_2_O_2_ and non-specific staining blocking with 10% goat serum. Then these sections were incubated with primary antibodies overnight at 4°C and treated for 30 min at 37°C with second antibody. The sections were staining for 2 min by using DAB and rinsed off in deionized water to terminate DAB reaction. Then using traditional method to evaluate under the optical microscope. The protein expression was calculated by German immunohistochemical score (GIS). Percentage of positive cells was graded as 0 (negative), 1 (up to 10%), 2 (11-50%), 3 (51-80%), or 4 (>80% positive cells) and staining intensity as 0 (no staining), 1 (weak), 2 (moderate), or 3 (strong). The final immunoreactive GIS was defined as the multiplication of both grading results (percentage of positive cells * staining intensity) (**Supplementary Table 2**).

### Statistical Analysis

The statistical data is consolidated and implemented by R-4.0.2. Continuous variables between two groups were compared using the unpaired Student t-test and Mann-Whitney U test for parametric data and non-parametric data, respectively. Survival curves were constructed using the Kaplan-Meier method, and the differences between the survival curves were examined by the log-rank test. Univariate Cox proportional hazards regressions were applied to estimate the individual hazard ratio (HR) for overall survival (OS). The NEC score of independent of various clinical features was performed by the univariate and multivariate Cox regression analysis. The receiver operating characteristic curve and the area under the curve (AUC) were calculated to the prediction accuracy of NEC score. All reported P values were two bsided and P<0.05 was considered statistically significant.

## Results

### Defining of the Expression, Prognostic, and Genetic Mutation Landscape of Necroptosis Related Genes in HCC

**Figure 1:**
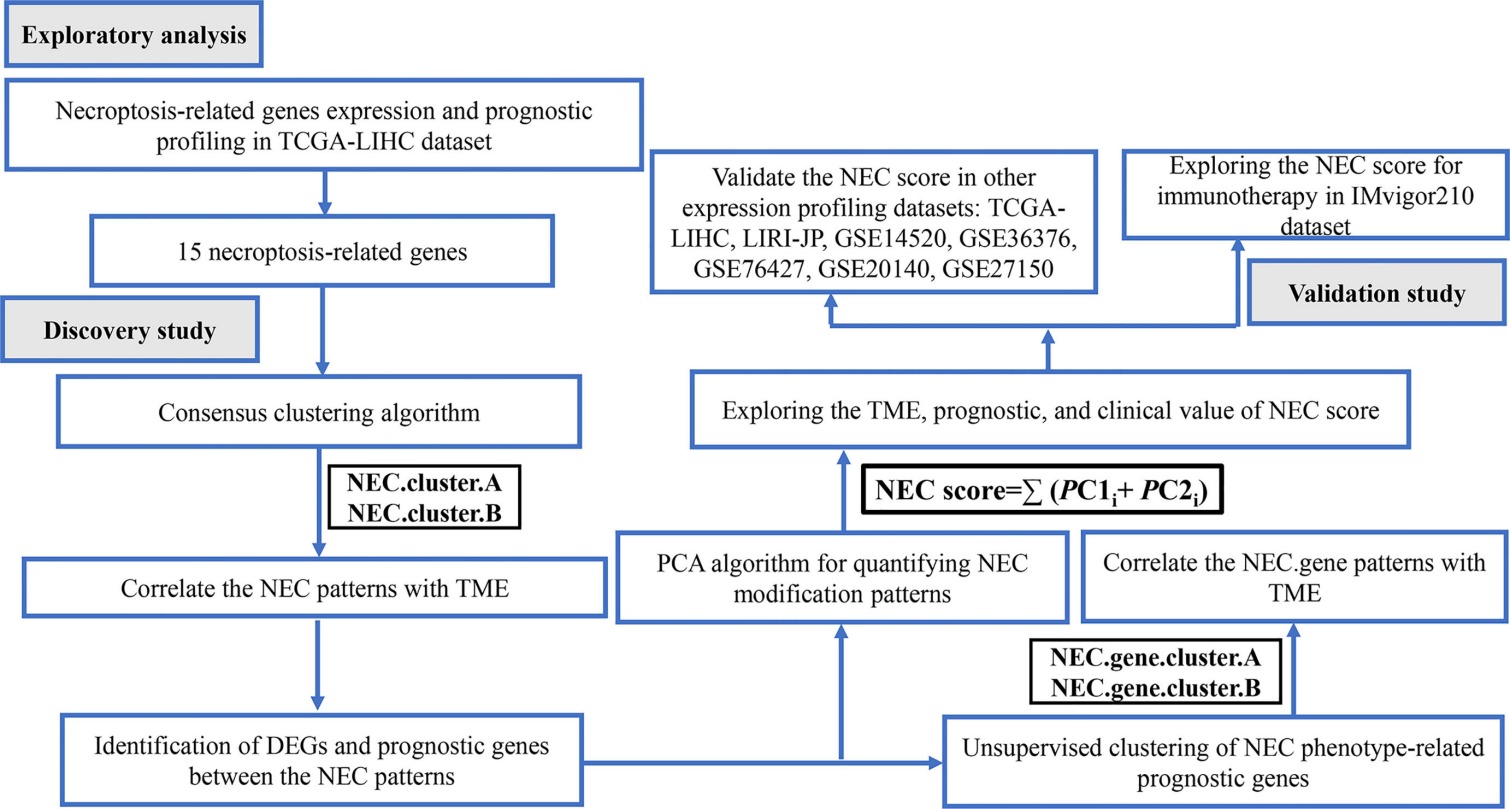
Flow chart of the study.

**Figure 2:**
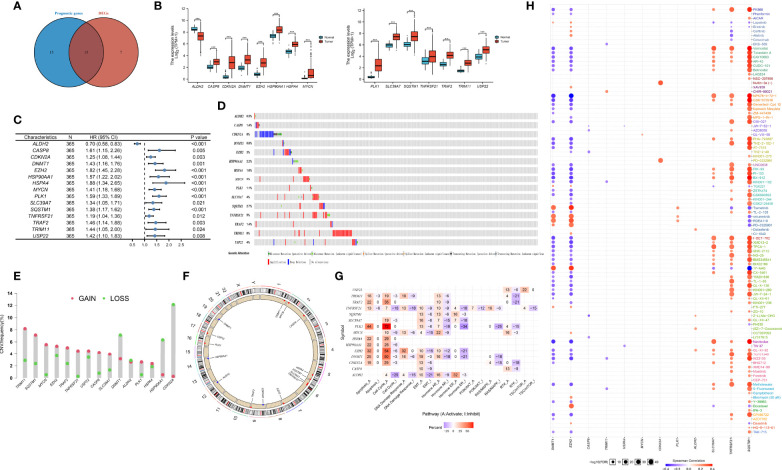
Defining of the expression, prognostic, and genetic mutation landscape of necroptosis related genes in TCGA-LIHC cohort. **(A)** Venn diagram shown 15 differentially expressed necroptosis related genes that were correlated with OS. **(B)** The illustration shown the expression of 15 differentially expressed necroptosis related genes between paired normal (blue) and HCC (red) tissues (Student’s t test). The asterisks represented the statistical p-value (*P < 0.05; **P < 0.01; ***P < 0.001). **(C)** Forest plots shown the results of the univariate Cox regression between 15 differentially expressed necroptosis related genes and overall survival in HCC. **(D)** The mutation frequency of 15 necroptosis related genes in 365 patients with HCC from TCGA-LIHC cohort. Each column represented individual patients. The number on the left indicated the mutation frequency in each gene. **(E)** The CNV variation frequency of 15 necroptosis related genes in TCGA-LIHC cohort. The height of the column represented the alteration frequency. The blue dot represent loss frequency; The red dot represent gain frequency. **(F)** The location of CNV alteration of 15 necroptosis related genes on 23 chromosomes using TCGA-LIHC cohort. **(G)** The illustration shown the relationship between cancer related pathways and 15 necroptosis related genes. **(H)** The illustration shown the relationship between drug sensitivity and 15 necroptosis related genes in GDSC database.

The flowchart of the detailed identification are shown in **Figure 1**. In order to obtain differentially expressed necroptosis-related genes in HCC, the expression data of HCC were collected from the TCGA-LIHC dataset. The results suggested that 19 necroptosis related genes (*CASP8*, *HSP90AA1*, *RNF31*, *NR2C2*, *HSPA4*, *USP22*, *TNFRSF21*, *SLC39A7*, *TSC1*, *SQSTM1*, *TRIM11*, *TRAF2*, *DNMT1*, *EZH2*, *LEF1*, *PLK1*, *MYCN*, *CDKN2A*, and *TERT*) were upregulated while 3 necroptosis related genes (*ID1*, *ALDH2*, and *BACH2*) were downregulated in HCC versus paired non-tumor tissues (|log2FC|>1 and adjusted P <0.05) (**Supplementary Table 3**). Next, to determine the prognostic of necroptosis related genes in HCC, 365 cases of HCC with sufficient survival data were analyzed using the univariate Cox analysis. The result showed that high expression of 26 necroptosis-related genes had a shorter OS than the low expression, while 4 necroptosis related genes had a better OS than the low expression (**Supplementary Table 4**). Finally, 15 differentially expressed necroptosis-related genes that were correlated with OS (**Figure 2A**). **Figures 2B, C** showed the expression and prognostic of 15 necroptosis related genes in HCC.

To explore the genetic mutation of these genes in HCC, we assessed the incidence of copy number variations (CNVs) and somatic mutations. We found that *CDKN2A* (8%) and *TRIM11* (8%) showed the highest mutation frequency followed by *TNFRSF21* (**Figure 2D**). Further analysis of CNV alteration frequency showed that *TRIM11*, *SQSTM1*, *MYCN*, and *EZH2* had a high frequency of CNV amplification, whereas *DNMT1*, *HSP90AA1*, and *CDKN2A* mainly showed CNV depletion (**Figure 2E**). The location of CNV alteration of the modification regulators on the chromosome is shown in **Figure 2F**. We found that most of necroptosis-related genes (except *DNMT1*, *HSP90AA1*, and *CDKN2A*) with a high frequency of CNV gain were highly expressed in HCC patients, suggesting that CNVs may be a potential contributor to the regulation of the expression of necroptosis-related genes.

Next, we assessed the correlation between the expression of necroptosis-related genes and cancer-related pathways (apotoptosis, cell cycle, DNA damage resposone, EMT, PI3K/AKT, RTK). The results showed that the expression of necroptosis-related genes was positively correlated with activite cancer-related pathways (**Figure 2G**). Research on drug sensitivity linked the drug response to possible biological effects through analysis of gene expression profiles. To gain further insight into the effects of the necroptosis-related genes on drug sensitivity, we evaluated the correlation between necroptosis-related genes and drug sensitivity, which were downloaded from the GDSC and CTRP database. The results showed the correlation and significance of most of drugs with the highest correlation coefficients in GDSC (**Figure 2H**) and CTRP databases (**Supplementary Figure 1**). These results implied that necroptosis-related genes are associated with drug sensitivity, and necroptosis-related genes might have potential applications in the development of novel chemotherapeutic drugs.

### Consensus Clustering of 15 Necroptosis-Related Genes Identified Two Clusters in HCC

**Figure 3:**
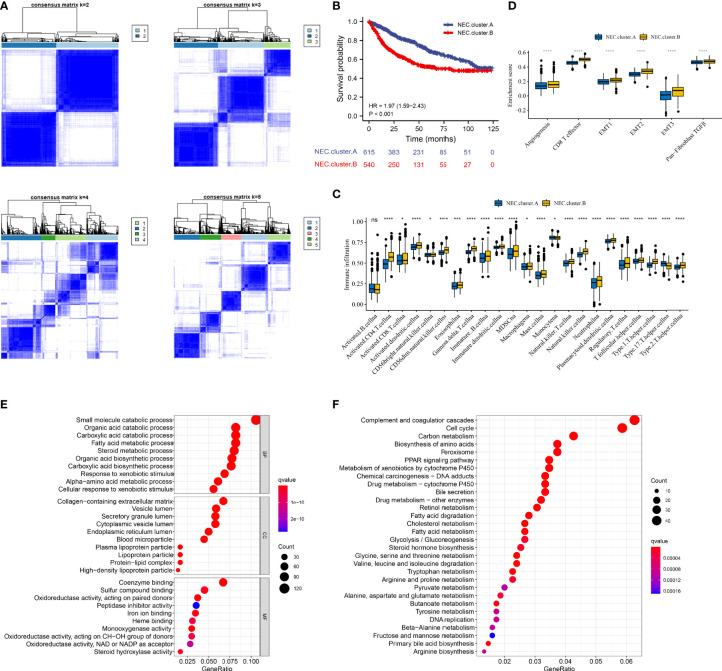
Necroptosis subtypes and biological characteristics of two distinct subtypes of samples divided by consistent clustering. **(A)** Unsupervised consensus clustering for 1155 HCC patients in a meta cohort (TCGA-LIHC, LIRI-JP, GSE14520, GSE36376, and GSE76427). **(B)** Survival analyses for the two NEC.clusters based on 1155 patients with HCC from five cohorts (TCGA-LIHC, LIRI-JP, GSE14520, GSE36376, and GSE76427) including 615 cases in NEC.cluster.A (blue), and 540 cases in NEC.cluster.B (red). Kaplan-Meier curves with Log-rank p value < 0.001 showed a significant survival difference among two modification patterns. The NEC.cluster.A showed significantly better overall survival than the NEC.cluster.B. (C) The illustration shown the abundance of 23 immune infiltrating cell in NEC.cluster.A (blue) andNEC.cluster.B (yellow). The upper and lower ends of the boxes represented the interquartile range of values. The lines in the boxes represented median value, and black dots showed outliers (Student’s t test). The asterisks represented the statistical p-value (*P < 0.05; **P < 0.01; ***P < 0.001; ****P < 0.0001). **(D)** Differences in stroma-activated pathways including EMT, TGF beta, and angiogenesis pathways among two NEC clusters (Student’s t test). The asterisks represented the statistical p-value (*P < 0.05; **P < 0.01; ***P < 0.001; ****P < 0.0001). **(E)** GO enrichment analysis. **(F)** KEGG enrichment analysis. The illustration was used to visualize these biological processes, where red represented activated pathways and blue represented inhibited pathways.

Next, 5 datasets (TCGA-LIHC, LIRI-JP, GSE14520, GSE36376, and GSE76427) with available OS data and clinical information were enrolled into one meta-cohort (N=1155). Based on 15 necroptosis-related genes in HCC, we conducted consensus clustering analysis to differentiate HCC patients. Two clusters (NEC.cluster.A and NEC.cluster.B) were suggested as the optimal clustering stability based on the similarity displayed by 15 necroptosis-related genes expression (**Figure 3A**, **Supplementary Table 5**). Interestingly, patients in NEC.cluster.B had a significantly shorter OS than in NEC.cluster.A (P< 0.001; **Figure 3B**). We then explored the difference in immune microenvironment of two clusters. **Figure 3C** showed the immune cell infiltration landscape in two clusters of HCC, which demonstrated that NEC.cluster.B was correlated with high abundance of immune infiltration levels (**Figure 3C**, **Supplementary Table 6**). Immune cells remained in the stroma around tumor cell nests rather than penetrating their parenchyma. Stroma activation in TME is thought to be T cell inhibitory (32). The results showed that patients in NEC.cluster.B group had a significantly higher stroma activity than in NEC.cluster.A group (**Figure 3D**).

### Identification of DEGs and Prognostic Genes Between the NEC Patterns

In order to explore the differential expression of genes between NEC.cluster.A and NEC.cluster.B. Based on the cutoff criterion of |log FC|≥1.0 and adj.P<0.05, there are 7987 DEGs between NEC.cluster.A and NEC.cluster.B (**Supplementary Table 4**). Next, to determine the prognostic of DEGs in HCC, 1155 cases of HCC with sufficient survival data were analyzed using the univariate Cox analysis. Finally, 4000 DEGs that were correlated with OS (**Supplementary Table 7**). Next, we performed GO and KEGG enrichment analysis to understand the potential functions of these 4000 genes. As a consequently, these genes showed widespread association with catabolic process, metabolic process in GO analysis (**Figure 3E**). Furthermore, these genes showed widespread association with cell cycle and metabolism in KEGG pathway analysis (**Figure 3F**).

**Figure 4:**
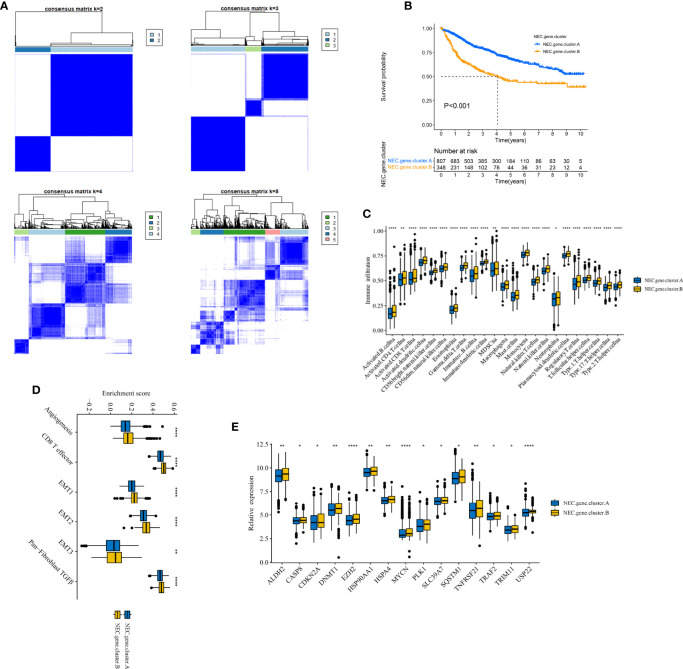
Identification of DEGs and prognostic genes between the NEC patterns. **(A)** Unsupervised consensus clustering based on prognostic NEC-related differentially expressed genes to classify patients into two groups termed NEC.gene.cluster.A, NEC.gene.cluster.B. **(B)** Survival analyses for the two NEC.gene.clusters based on 1155 patients with HCC from five cohorts (TCGA-LIHC, LIRI-JP, GSE14520, GSE36376, and GSE76427) including 807 cases in NEC.gene.cluster.A (blue), and 348 cases in NEC.gene.cluster.B (yellow). Kaplan-Meier curves with Log-rank p value < 0.001 showed a significant survival difference among two NEC.gene.clusters. The NEC.gene.cluster.A showed significantly better overall survival than the NEC.cluster.B. (C) The illustration shown the abundance of 23 immune infiltrating cell in NEC.gene.cluster.A (blue) and NEC.gene.cluster.B (yellow). The upper and lower ends of the boxes represented the interquartile range of values. The lines in the boxes represented median value, and black dots showed outliers (Student’s t test). The asterisks represented the statistical p-value (*P < 0.05; **P < 0.01; ***P < 0.001; ****P < 0.0001). **(D)** The illustration shown the different between stroma-activated pathways including EMT, TGF beta, and angiogenesis pathways in NEC.gene.cluster.A (blue) and NEC.gene.cluster.B (yellow). The upper and lower ends of the boxes represented the interquartile range of values. The lines in the boxes represented median value, and black dots showed outliers (Student’s t test). The asterisks represented the statistical pvalue (*P < 0.05; **P < 0.01; ***P < 0.001; ****P < 0.0001). **(E)** The illustration shown the expression of 15 necroptosis related genes between NEC.gene.cluster.A (blue) and NEC.gene.cluster.B (yellow) (Student’s t test). The upper and lower ends of the boxes represented interquartile range of values. The lines in the boxes represented median value, and black dots showed outliers. The asterisks represented the statistical p-value (*P < 0.05; **P < 0.01; ***P < 0.001; ****P < 0.0001).

Next, based on 4000 genes in HCC, we conducted consensus clustering analysis to differentiate HCC patients. Two clusters (NEC.gene.cluster.A and NEC.gene.cluster.B) were suggested as the optimal clustering stability based on the similarity displayed by 4000 genes expression (**Figure 4A**, **Supplementary Table 8**). Moreover, patients in NEC.gene.cluster.B group had a significantly shorter OS than those in NEC.gene.cluster.A group (P< 0.001; **Figure 4B**). We then explored the difference in immune microenvironment of this two clusters. **Figure 4C** showed the immune cell infiltration landscape in two clusters of HCC, which demonstrated that NEC.gene.cluster.B was correlated with high abundance of immune infiltration levels (**Supplementary Table 9**). Moreover, patients in NEC.gene.cluster.B group had a significantly higher stroma activity than in NEC.gene.cluster.A group (**Figure 4D**), and the significant overexpressions of 15 necroptosis-related genes in NEC.gene.cluster.B were observed relative to NEC.gene.cluster.A (**Figure 4E**). The above results proved the effectiveness and stability of NEC patterns.

### Construction of NEC Score

**Figure 5:**
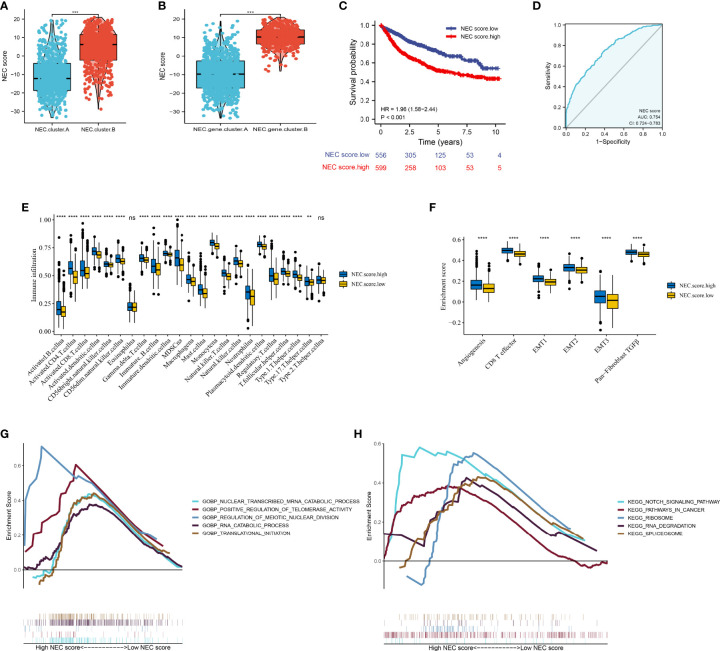
Construction of NEC score. **(A)** Differences in NEC score among two NEC.clusters in meta cohort (Student’s t test). **(B)** Differences in NEC score among two NEC.gene.clusters in meta cohort (Student’s t test). **(C)** Kaplan-Meier curves for high and low NEC score patient groups. **(D)** The predictive value of NEC score in meta cohort. **(E)** The abundance of each TME infiltrating cell in high and low NEC score groups (Student’s t test). The upper and lower ends of the boxes represented the interquartile range of values. **(F)** Differences in stroma-activated pathways including EMT, TGF beta, and angiogenesis pathways among high and low NEC score groups (Student’s t test). **(G)** GSEA GO identified high and low NEC score groups related signaling pathways in HCC. **(H)** GSEA KEGG identified high and low NEC score related signaling pathways in HCC.

Next, to quantify the NEC index of each HCC patient, we constructed a scoring system using the PCA method based on NEC phenotype-related prognostic genes (**Supplementary Table 10**). The results showed that compare with NEC.cluster.A (**Figure 5A**), and NEC score was significantly higher in NEC.cluster.B, and compare with NEC.gene.cluster.A, NEC score was significantly higher in NEC.gene.cluster.B (**Figure 5B**). In addition, patients with high NEC score had a significantly shorter OS than those with low NEC score (P< 0.001; **Figure 5C**), and AUC of the ROC was 0.754 (**Figure 5D**). We then explored the difference in immune microenvironment of this two groups. **Figure 4C** showed the immune cell infiltration landscape in two groups of HCC, which demonstrated that high NEC score group was correlated with high abundance of immune infiltration levels (**Figure 5E**, **Supplementary Table 11**). Moreover, patients in high NEC score group had a significantly higher stroma activity than in low NEC score group (**Figure 5F**). Next, GSEA was performed to explore HCC involved signaling pathways between two groups. The results indicated that the high NEC score group was more likely to be enriched in RNA translation and cancer-specific pathways, including translational initiation, notch signaling pathway, ribosome, and spliceosome (**Figures 5G, H**).

### Further Survival Analysis of the NEC Score

**Figure 6:**
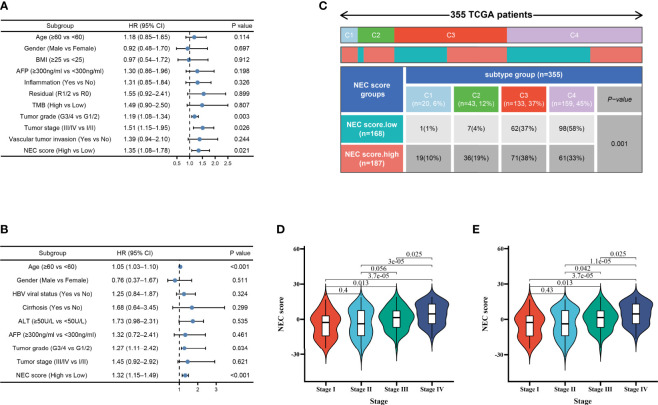
Independent prognostic analysis of NEC score. **(A)** Multivariate Cox regression analysis for NEC score in TCGA cohort shown by the forest plot. **(B)** Multivariate Cox regression analysis for NEC score in GSE14520/NCI cohort shown by the forest plot. **(C)** Differences in NEC score between immune subtypes (C1: wound healing; C2: IFN-gamma dominant; C3: inflammatory; C4: lymphocyte depleted) **(D)** Differences in NEC score between different stage in TCGA cohort (Student’s t test). **(E)** Differences in NEC score between different stage in GSE14520/NCI cohort (Student’s t test).

Considering clinical characters, we then performed univariate and multivariate analysis and identified NEC score as independent factors impacting on HCC patients’ prognosis in TCGA cohort (HR 1.35; 95% CI 1.08, 1.78; P=0.021) (**Figure 6A**) and GSE14520/NCI cohort (HR 1.32; 95% CI 1.15, 1.49; P<0.001) (**Figure 6B**). To detect NEC score in TCGA immune subtypes, we downloaded the TCGA immune subtypes of HCC patients from the TCGA database, and we found patients with high NEC score were characterized wound healing, IFN-gamma dominant, and inflammatory phenotype (**Figure 6C**). We also detect the association between NEC score and clinicopathological features of patients with HCC in TCGA cohort and GSE14520/NCI cohort. We found that the NEC score increased with an increase in the HCC stage, which translates to a worse prognosis in TCGA cohort (**Figure 6D**) and GSE14520/NCI cohort (**Figure 6E**).

### Validation of NEC Score in Multiple Cohorts

**Figure 7:**
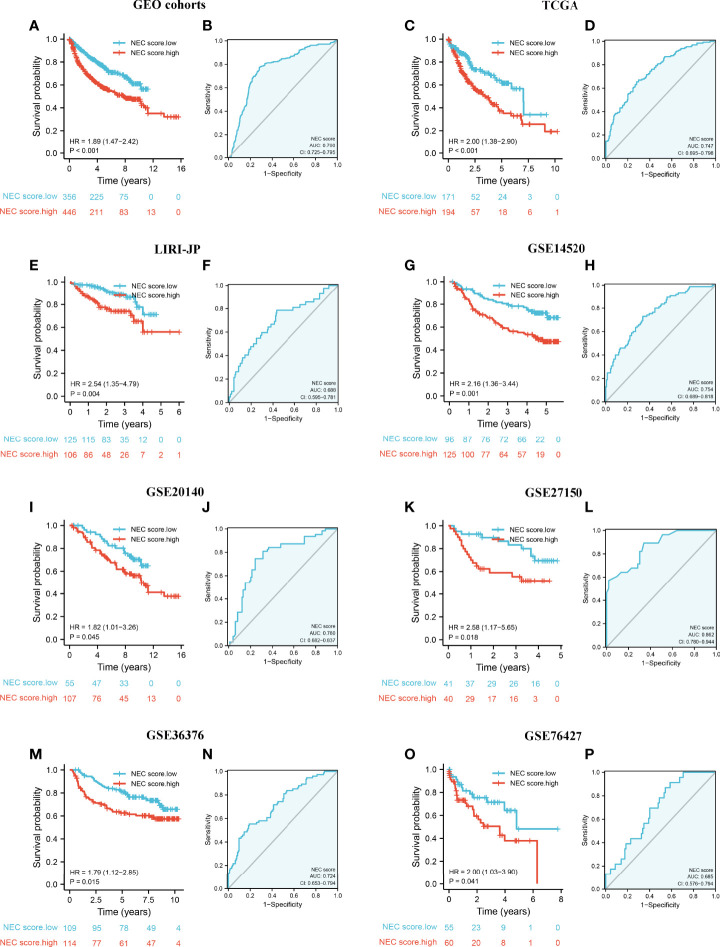
External validation of NEC score model. **(A)** Kaplan-Meier curves for high and low NEC score patient groups in 5 GEO datasets (GSE14520/NCI, GSE36376, GSE76427, GSE20140, and GSE27150). **(B)** The predictive value of NEC score in 5 GEO dataset. **(C)** Kaplan-Meier curves for high and low NEC score patient groups in TCGA-LIHC. **(D)** The predictive value of NEC score in TCGA-LIHC. **(E)** Kaplan-Meier curves for high and low NEC score patient groups in LIRI-JP. **(F)** The predictive value of NEC score in LIRI-JP. **(G)** Kaplan-Meier curves for high and low NEC score patient groups in GSE14520/NCI. **(H)** The predictive value of NEC score in GSE14520/NCI. **(I)** Kaplan-Meier curves for high and low NEC score patient groups in GSE20140. **(J)** The predictive value of NEC score in GSE20140. **(K)** Kaplan-Meier curves for high and low NEC score patient groups in GSE27150. **(L)** The predictive value of NEC score in GSE27150. **(M)** Kaplan-Meier curves for high and low NEC score patient groups in GSE36376. **(N)** The predictive value of NEC score in GSE36376. **(O)** Kaplan-Meier curves for high and low NEC score patient groups in GSE76427. **(P)** The predictive value of NEC score in GSE76427.

To validate the stability of the NEC score model, we applied the NEC score established in the meta cohort to 7 external independent GEO or TCGA HCC datasets. The result showed that patients with a high NEC score had a poor prognosis in all GEO dataset (HR 1.89; 95% CI 1.47, 2.42; P<0.001) (**Figure 7A**), and the AUC of the ROC was 0.760 (**Figure 7B**). In addition, patients with a high NEC score had a poor prognosis in all TCGA dataset (HR 2.00; 95% CI 1.38, 2.90; P<0.001) (**Figure 7C**), LIRI-JP dataset (HR 2.54; 95% CI 1.35, 4.79; P=0.004) (**Figure 7E**), GSE14520 (HR 2.16; 95% CI 1.36, 3.44; P-0.001) (**Figure 7G**), GSE20140 (HR 1.82; 95% CI 1.01, 3.26; P=0.045) (**Figure 7I**), GSE27150 (HR 2.58; 95% CI 1.17, 5.65; P=0.018) (**Figure 7K**), GSE36376 (HR 1.79; 95% CI 1.12, 2.85; P=0.015) (**Figure 7M**), and GSE76427 (HR 2.00; 95% CI 1.03, 3.90; P=0.041) (**Figure 7O**), and the AUC of the ROC was 0.747 in TCGA dataset (**Figure 7D**), 0.688 in LIRI-JP dataset (**Figure 7F**), 0.754 in GSE14520 (**Figure 7H**), 0.760 in GSE20140 (**Figure 7J**), 0.862 in GSE27150 (**Figure 7L**), 0.724 in GSE36376 (**Figure 7N**), and 0.685 in GSE76427 (**Figure 7P**). These results demonstrated that NEC score is an effective and stable model and can predict the prognosis of patients with HCC.

### Association of NEC Score With Immunotherapy

**Figure 8:**
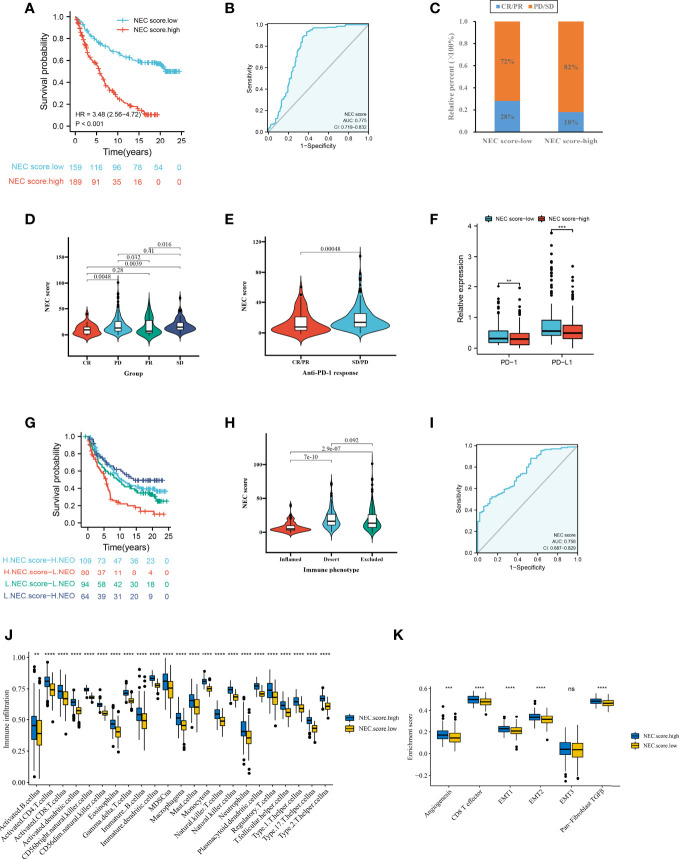
NEC score in the role of anti-PD-1/L1 immunotherapy. **(A)** Survival analyses for low and high NEC score patient groups in the anti-PD-L1 immunotherapy cohort using Kaplan-Meier curves (IMvigor210 cohort). **(B)** The predictive value of NEC score in IMvigor210 cohort. **(C)** The proportion of patients with response to PD-L1 blockade immunotherapy in low or high NEC score groups. SD, stable disease; PD, progressive disease; CR, complete response; PR, partial response. **(D)** Differences in NEC score among distinct anti-PD-1 clinical response groups (Student’s t test). **(E)** Distribution of NEC score in distinct anti-PD-L1 clinical response groups (Student’s t test). **(F)** Differences in checkpoint expression between low and high NEC score groups (Student’s t test). The asterisks represented the statistical p-value (*P < 0.05; **P < 0.01; ***P < 0.001). **(G)** Survival analyses for patients receiving anti-PD-L1 immunotherapy stratified by both NEC score and neoantigen burden using Kaplan-Meier curves. **(H)** Differences in NEC score between immune subtypes (Student’s t test). **(I)** The predictive value of the quantification of NEC patterns in patients treated with anti-PD-1/L1 immunotherapy . **(J)** The abundance of each TME infiltrating cell in high and low NEC score groups (Student’s t test). **(K)** Differences in stromaactivated pathways and abundance of regulatory T cells (considered as immune suppression) between low and high NEC score groups in anti-PD-L1 immunotherpy cohort (Student’s t test).

Firstly, after consensus clustering analysis of these samples by ConsensusClusterPlus algorithm base on 15 necroptosis-related genes in IMvigor210 cohort, it revealed that the results were most stable at K=2. Then, 1278 DEGs that were correlated with OS were identified (**Supplementary Table 12**). Finally, we constructed a NEC score base on 1278 genes (**Supplementary Table 13**). The result showed that patients with a high NEC score had a poor prognosis in IMvigor210 dataset (HR 3.48; 95% CI 2.56, 4.72; P<0.001) (**Figure 8A**), and the AUC of the ROC was 0.775 (**Figure 8B**). We further detect the association between NEC score and immunotherapy. We found that the proportion of CR/PR patients in low NEC score group was significantly higher than that in high NEC score group (**Figure 8C**, P < 0.05), and the NEC score in CR/PR patients was significantly lower than that of SD/PD (**Figures 8D, E**, **Table 2**, P<0.05). Moreover, the low NEC score group had a high checkpoint expression levels. Above results can partly explain the poor prognosis in high NEC score group.

**Table 2:** Clinical characteristics of the bladder cancer patients in IMvigor210 (mUC) cohort used in this study.

IMvigor210 cohort	Low (n = 159)	High (n = 189)	Total (n = 348)
**Vital status**			
Alive	57 (35.8%)	59 (31.2%)	116 (33.3%)
Dead	102 (64.2%)	130 (68.8%)	232 (66.7%)
**Gender**			
Female	28 (17.6%)	48 (25.4%)	76 (21.8%)
Male	131 (82.4%)	141 (74.6%)	272 (78.2%)
**Overall response**			
CR	13 (9.4%)	12 (7.5%)	25 (8.4%)
PR	26 (18.7%)	17 (10.7%)	43 (14.4%)
SD	47 (33.8%)	16 (10.1%)	63 (21.1%)
PD	53 (38.1%)	114 (71.7%)	167 (56.0%)
**Binary response**			
CR/PR	39 (28.1%)	29 (18.2%)	68 (22.8%)
SD/PD	100 (71.9%)	130 (81.8%)	230 (77.2%)
**Enrollment IC**			
IC0	44 (27.7%)	55 (29.1%)	99 (28.4%)
IC1	64 (40.3%)	68 (36.0%)	132 (37.9%)
IC2	51 (32.1%)	66 (34.9%)	117 (33.6%)
**IC level**			
IC0	42 (26.9%)	55 (29.1%)	97 (28.0%)
IC1	64 (41.0%)	68 (36.0%)	132 (38.0%)
IC2+	50 (32.1%)	66 (34.9%)	118 (34.0%)
**TC Level**			
TC0	131 (82.9%)	144 (76.2%)	275 (79.3%)
TC1	7 (4.4%)	15 (7.9%)	22 (6.3%)
TC2+	20 (12.7%)	30 (15.9%)	50 (14.4%)
**Immune phenotype**			
Desert	22 (19.5%)	54 (31.6%)	76 (26.8%)
Excluded	52 (46.0%)	82 (48.0%)	134 (47.2%)
Inflamed	39 (34.5%)	35 (20.5%)	74 (26.1%)
**TCGA cluster**			
I	53 (33.3%)	65 (34.3%)	118 (33.9%)
II	51 (32.1%)	44 (23.3%)	95 (27.3%)
III	28 (17.6%)	41 (21.7%)	69 (19.8%)
IV	27 (17.0%)	39 (20.6%)	66 (19.0%)

Additionally, to compare the prognostic analysis effect of NEC score in patients who had a high or low neoantigen burden, survival analysis was also performed on patients in the IMvigor210 cohort. We revealed that among both patients who had a high or low neoantigen burden, patients with a high NEC score showed lower overall survival (**Figure 8G**, P<0.05), and the AUC of the ROC was 0.758 (**Figure 8H**). To identify the association of the immune subtype with the NEC score, we traced each sample from the immune subtype to the NEC score. As shown in **Figure 8I**, patients with the inflamed subtype were assigned a low NEC score, which was consistent with better prognosis in these groups. We then explored the difference in immune microenvironment of this two groups. **Figure 8J** showed the immune cell infiltration landscape in two groups, which demonstrated that high NEC score group was correlated with high abundance of immune infiltration levels (**Figure 8J**). Moreover, patients in high NEC score group had a significantly higher stroma activity than in low NEC score group (**Figure 8K**).

**Figure 9:**
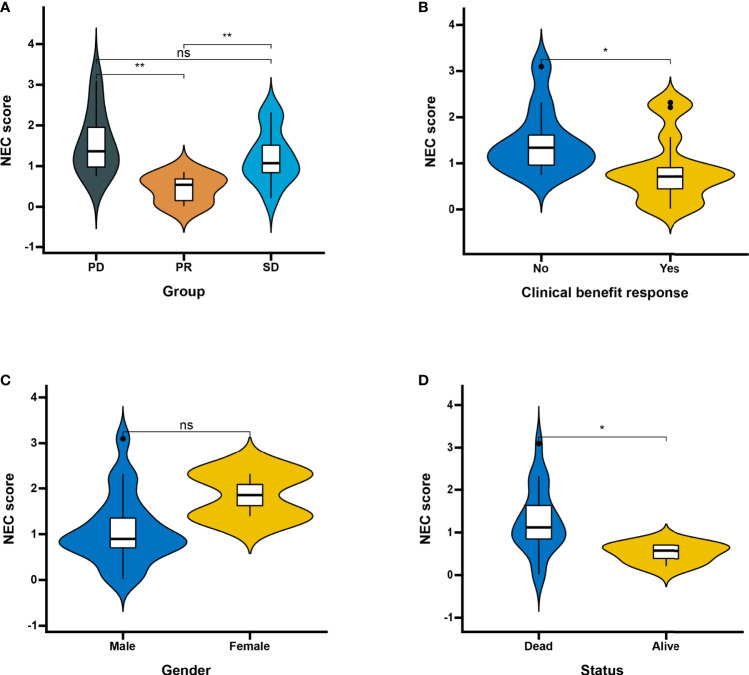
Patient characteristics and NEC score of HCC treated with anti-PD-1 immmunotherapy. **(A)** Distribution of NEC score in distinct anti-PD-L1 clinical response groups. SD, stable disease; PD, progressive disease; PR, partial response (Student’s t test). The asterisks represented the statistical p-value (**P < 0.01). **(B)** Differences in NEC score between clinical response groups (Student’s t test). The asterisks represented the statistical p-value (*P < 0.05). **(C)** Differences in NEC score between gender groups (ns: not significant). **(D)** Differences in NEC score between status groups (Student’s t test). The asterisks represented the statistical p-value (*P < 0.05).

Next, we used a HCC immune cohort (GSE140901) to detect the relationship between NEC score and immunotherapy. NEC score also varied statistically in the PR, SD, and PD groups, NEC score was notably lower in PR group than PD or SD groups (**Figure 9A**). We also discovered that NEC score was notably lower in clinical benefit response group than no clinical benefit response group (**Figure 9B**). The abovere sults suggests that NEC score was sensitivity to immunotherapy. We also compared the differential level of NEC score in different subgroups stratified by gender and status. As shown in **Figures 9C, D**, NEC score was no significantly betweent male and female, however, NEC score was significantly higher in dead group than in alive group.

### Validation the Expression of 15 Necroptosis-Related Genes by IHC

**Figure 10:**
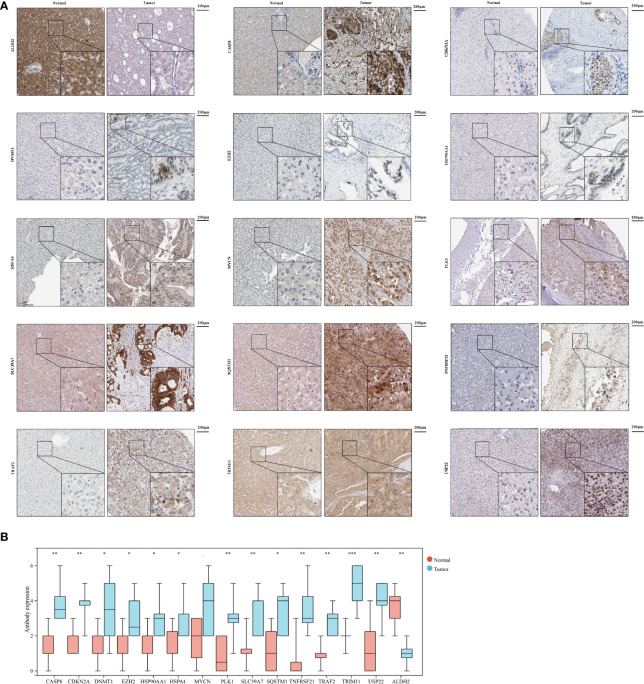
The expression of 15 necroptosis genes in HCC by IHC. **(A)** IHC staining of 15 necroptosis genes in HCC and normal tissues. **(B)** Statistic data of IHC analysis (N = 8). (*P < 0.05; **P < 0.01; ***P < 0.001).

To further validate necroptosis-related genes expression in HCC, IHC was used to measure the expression level of necroptosis-related genes in HCC (N=8), and the result showed that compared with normal group, the CASP8, CDKN2A, DNMT1, EZH2, HSP90AA1, HSPA4, MYCN, PLK1, SLC39A7, SQSTM1, TNFRSF21, TRAF2, TRIM11, and USP22 level were significantly higher in HCC group, while ALDH2 was downregulated in HCC versus normal tissues (**Figures 10A, B**).

## Discussion

Necroptosis was associated with tumor cell migration and invasion regulation (33). As a subform of programmed cell death, necroptosis was suggested as a promising approach to eliminating cancer cell (34). To elucidate the prognostic value and their correlation with complex tumor microenvironment in HCC will allow necroptosis to be exploited for the prognosis and therapy of HCC. In the context of clinical and RNA-seq data, we performed a retrospective analysis of histologically confirmed 1398 HCC patients. In this study, we identified 15 prognostic necroptosis related DEGs, and these necroptosis-related genes were positively correlated with activite cancer-related pathways, suggesting thses necroptosis-related genes play an important role in HCC. Based on 15 necroptosis-related genes, we classified HCC patients into 2 clusters. Next, we identified 4000 prognostic and DEGs between the 2 clusters. Subsequently, we constructed a NEC score based on NEC phenotype-related prognostic genes to quantify the necroptosis related subtypes of individual patients.

From a global perspective, NEC score is an effective and stable model and had a good performance in predicting the prognosis of HCC patients. Functional analysis demonstrated that high NEC score was more likely to be enriched in RNA translation and cancer-specific pathways, including translational initiation, notch signaling pathway, ribosome, and spliceosome. Interestingly, these functions or pathways were involved in necroptosis and tumor progression. RNA translation was showed to be involved in inflammation and cancer progression (35). Notch signaling pathway is closely related to the dysregulation of HCC cell apoptosis in the occurrence and development of HCC (36). Notch signaling pathway can inhibit or promote HCC cell apoptosis due to altered cellular and molecular environments (37). Notch signaling pathway regulates hepatocellular carcinoma cell apoptosis by “crosstalk” with other cell signaling pathways or directly affecting internal and external apoptosis pathways (38). Notch signaling pathway also play an indispensable part in mammalian immunity and cellular homeostasis (39).

NEC.cluster.B, NEC.gene.cluster.B, and high NEC score groups had a high abundance of immune infiltration, due to the stroma activity had been activated. Tumor microenvironment (TME) includes tumor cells, extracellular matrix (ECM), stromal cells, immune cells, peripheral blood vessels and signaling molecules (40). The resistance and insufficient effectiveness of various anti-tumor drugs have led to tumor recurrence and limited treatment, while the important role of TME in HCC has become a new breakthrough for treatment (41, 42). Immune-infiltrating cells seem to play a dual role in eliminating or promoting tumors in TME, which will advance our understanding of the signaling pathway and provide additional important targets for tumor immunotherapy (43). ECM is composed of basement membrane and intercellular stroma and is an important barrier to tumor metastasis. ECM is contained in a variety of substances, including tumor cells and other cells in the microenvironment of a large number of growth factors, cytokines and metalloproteinases, metabolism and tumor produce all kinds of acid, and the acid played a maintain tumor weak acid environment, can induce tumor ectomesenchymal transformation to the epithelial cells, and promote the role of hypoxia environment (43). Abnormal ECM not only acts as a cellular scaffold in TME, but also promotes tumor development through various secreted proteins (such as inhibiting collagen matrix deposition, promoting inflammation and angiogenesis) (44).

The positive expression rate of PD-L1 in HCC tumor cells was less than 10%, and no significant correlation was found between the positive expression of PD-L1 and the efficacy of immunotherapy in HCC patients in KEYNOTE-224 and CheckMate 040 studies (45, 46). Tumor mutation burden (TMB) is the second tumor concomitant diagnostic marker approved for clinical application, but the level of TMB for HCC is not significant compared with other tumors. However, mismatch repair and microsatellite instability only occur in 2%-3% of HCC patients (47). Therefore, the application value of current clinically applied biomarkers in HCC is limited. More and more studies have shown that necroptosis-related genes can be used as prognostic markers for cancer and other diseases. Meanwhile, NEC score established by NEC phenotype-related prognostic genes can effectively predict the immune efficacy of patients.

There are some limitations to our study. Firstly, the molecular mechanism of the core genes need to be further verified by experiments. The underlying relationship between NEC score and immunotherapy awaits follow-up studies.

In conclusion, we performed a comprehensive bioinformatics analysis for necroptosis-related genes and constructed a NEC score based on NEC phenotype-related prognostic genes. The established NEC score would contribute to predicting the prognosis of HCC patients and the response to anti-PD-1/L1 immunotherapy.

## Data Availability Statement

The original contributions presented in the study are included in the article/**Supplementary Material**. Further inquiries can be directed to the corresponding author.

## Ethics Statement

This study was approved by the Ethics Review Board of the Maoming People’s Hospital. The patients/participants provided their written informed consent to participate in this study.

## Author Contributions

SH designed the study and drafted the manuscript. SH and XY helped to draft the manuscript. XY conceived the study, participated in its design and coordination, and helped to draft the manuscript. JW, ML, XY, LW, ZZ, HM, and YC helped to revise the manuscript. YC performed statistical analysis. All authors contributed to the article and approved the submitted version.

## Funding

This work was supported by the Maoming Science and Technology Plan (grant nos. 2020KJZX013). This study was also supported by High-level Hospital Construction Research Project of Maoming People’s Hospital.

## Conflict of Interest

The authors declare that the research was conducted in the absence of any commercial or financial relationships that could be construed as a potential conflict of interest.

## Publisher’s Note

All claims expressed in this article are solely those of the authors and do not necessarily represent those of their affiliated organizations, or those of the publisher, the editors and the reviewers. Any product that may be evaluated in this article, or claim that may be made by its manufacturer, is not guaranteed or endorsed by the publisher.

## Supplementary Material

Supplementary Figure 1 The relationship between drug sensitivity and 15 necroptosis genes in CTRP database.

Supplementary Table 1 List of 76 necroptosis related genes used in this study

Supplementary Table 2 Information about the antibodies used in this study.

Supplementary Table 3 Differentially expressed apoptosis related genes between tumor and normal tissue in TCGA-LIHC dataset

Supplementary Table 4 The results of the univariate Cox regression analysis between gene expression and OS in TCGA-LIHC dataset

Supplementary Table 5 Unsupervised consensus clustering for 1155 HCC patients in a meta cohort (TCGA-LIHC, LIRI-JP, GSE14520, GSE36376, and GSE76427) base on 15 necroptosis related genes.

Supplementary Table 6 The abundance of each TME infiltrating cell betweent NEC.cluster.A and NEC.cluster.B groups.

Supplementary Table 7 List of 4000 NEC phenotype-related prognostic genes used in this study.

Supplementary Table 8 Unsupervised consensus clustering for 1155 HCC patients in a meta cohort (TCGA-LIHC, LIRI-JP, GSE14520, GSE36376, and GSE76427) base on 4000 NEC phenotype-related prognostic genes.

Supplementary Table 9 The abundance of each TME infiltrating cell betweent NEC.gene.cluster.A and NEC.gene.cluster.B groups.

Supplementary Table 10 Summary of detailed of NEC score for each HCC patients.

Supplementary Table 11 The abundance of each TME infiltrating cell betweent NEC score.high and NEC score.low groups.

Supplementary Table 12 List of 1278 NEC phenotype-related prognostic genes used in this study in IMvigor210 cohort.

Supplementary Table 13 Summary of detailed clinical information and apoptosisscore in IMvigor210 (mUC) cohort.
