# Supplementary figures and images for: Necroptosis-Related Genes Signatures Identified Molecular Subtypes and Underlying Mechanisms in Hepatocellular Carcinoma
Source: Front Oncol. 2022 Jul 13;12:875264. doi: 10.3389/fonc.2022.875264 (PMC9326098; doi:10.3389/fonc.2022.875264)

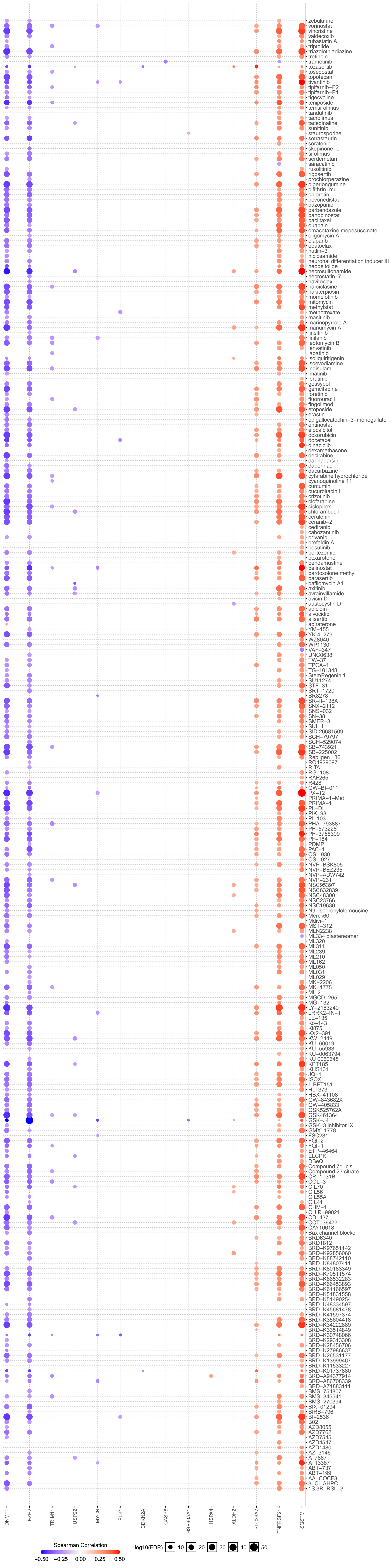

Supplement: Supplementary Figure 1 — The relationship between drug sensitivity and 15 necroptosis genes in CTRP database. [file DataSheet_1.pdf]
